# Supplementary material for: Nanocellulose from Cocoa Shell in Pickering Emulsions of Cocoa Butter in Water: Effect of Isolation and Concentration on Its Stability and Rheological Properties
Source: Polymers (Basel). 2023 Oct 19;15(20):4157. doi: 10.3390/polym15204157 (PMC10610805; doi:10.3390/polym15204157)
Supplement: Supplementary file 1 [file polymers-15-04157-s001.zip › Supplemetary materials.pdf]

Nanocellulose from Cocoa Shell in Pickering Emulsions of Cocoa Butter in Water: Effect of Isolation and Concentration on its Stability and Rheological Properties.

Catalina Gómez Hoyos[1,*] Luis David Botero[1]; Andrea Flórez-Caro[1], Jorge Andrés Velásquez-Cock[1] and Robin Zuluaga [2]

[1] Programa de Ingeniería en Nanotecnología, Universidad Pontificia Bolivariana, Circular 1 N_ 70-01, Medellín 050031, Colombia; luisd.botero@upb.edu.co (L.D.B.); andrea.florezc@upb.edu.co (A.F.-C.); jorgeandres.velasquez@upb.edu.co (J.A.V.-C.)

[2] Facultad de Ingeniería Agroindustrial, Universidad Pontificia Bolivariana, Circular 1 N_ 70-01, Medellín 050031, Colombia; robin.zuluaga@upb.edu.co

* Correspondence: catalina.gomezh@upb.edu.co
*Correspondence: catalina.gomezh@upb.edu.co

Supplementary information

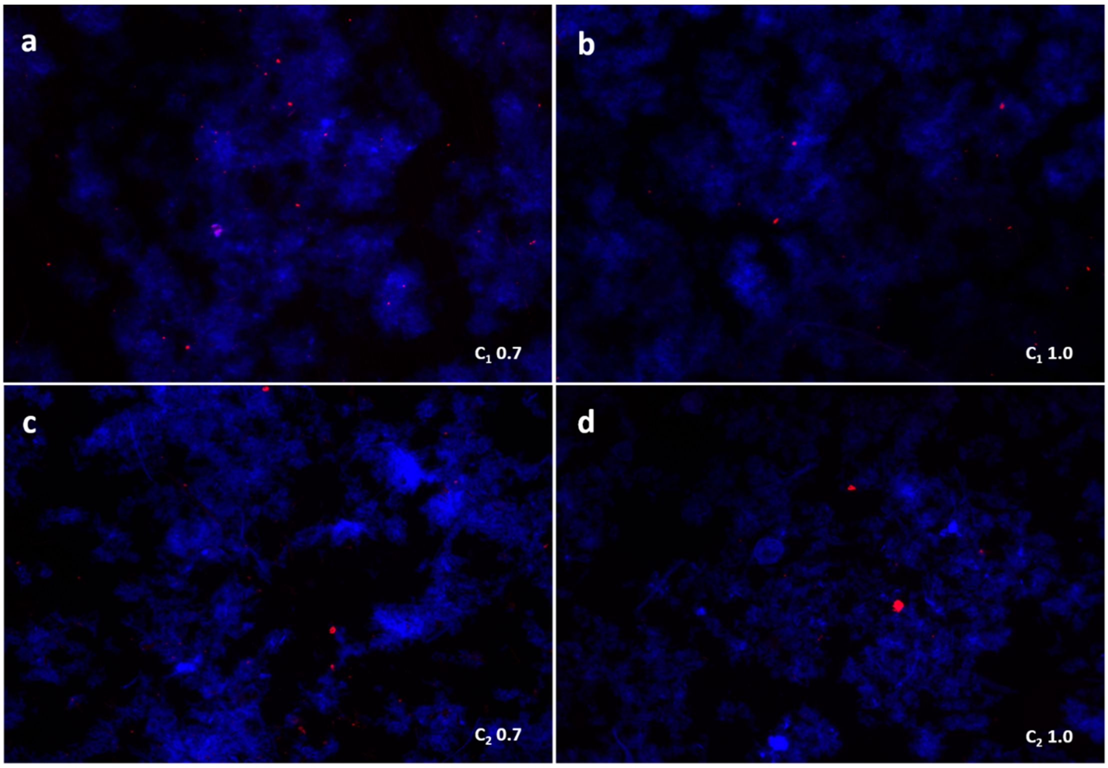

Figure S1. Fluorescent microscopy images of CNFs; (a, c) 0.7 wt%; (b, d) 1.0 wt%.Oil phase is shown in red, and the cellulose is shown in blue..

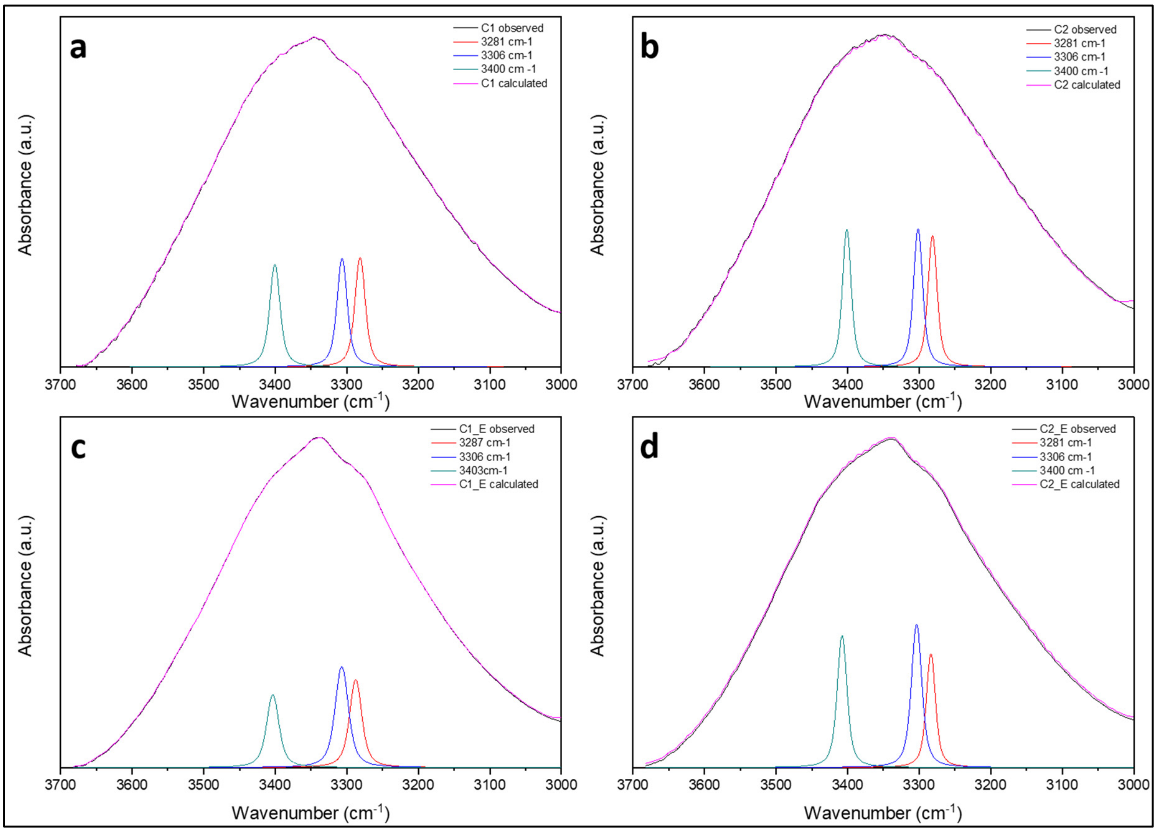

Figure S2. ATR-FTIR spectra deconvolution between 3700 and 3000 cm^{-1}. _E represents sample after removing extractives: (a, c) ATR-FTIR spectra deconvolution of C_1 samples; (b, d) ATR-FTIR spectra deconvolution of C_2 samples.
